# Supplementary material for: Pharmacological Gq targeting prevents asthmatic airway remodeling
Source: Mol Ther. 2025 Jul 23;33(10):5149–61. doi: 10.1016/j.ymthe.2025.07.032 (PMC12848170; doi:10.1016/j.ymthe.2025.07.032)
Supplement: Document S1. Figures S1–S3 [file mmc1.pdf]

YMTHE, Volume *33*

Supplemental Information

Pharmacological Gq targeting

prevents asthmatic airway remodeling

Jennifer M. Dietrich, Michaela Matthey, Annika Simon, Alexander Seidinger, Cynthia Koziol-White, Reynold A. Panettieri Jr., Bernd K. Fleischmann, and Daniela Wenzel

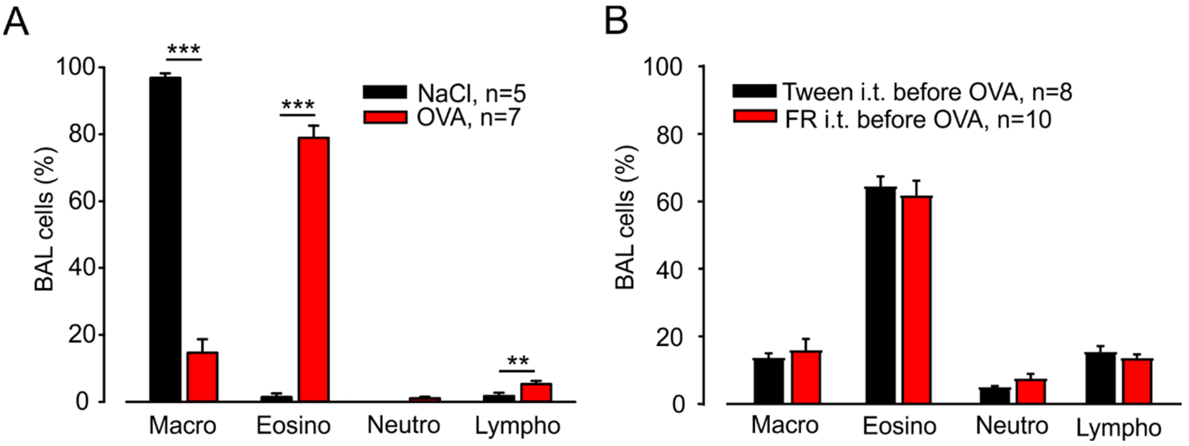

Figure S1. FR does not affects inflammatory cell counts in the OVA-induced mouse model of chronic asthma. A,B) Relative cell counts in BAL fluid for control and OVA-exposed Balb/c mice with chronic asthma (A), relative cell counts in BAL fluid for FR- or solvent-treated OVA-sensitized Balb/c mice (B), n represents the number of animals, ***P<0.001. A.B) Unpaired two-tailed student`s t-test.

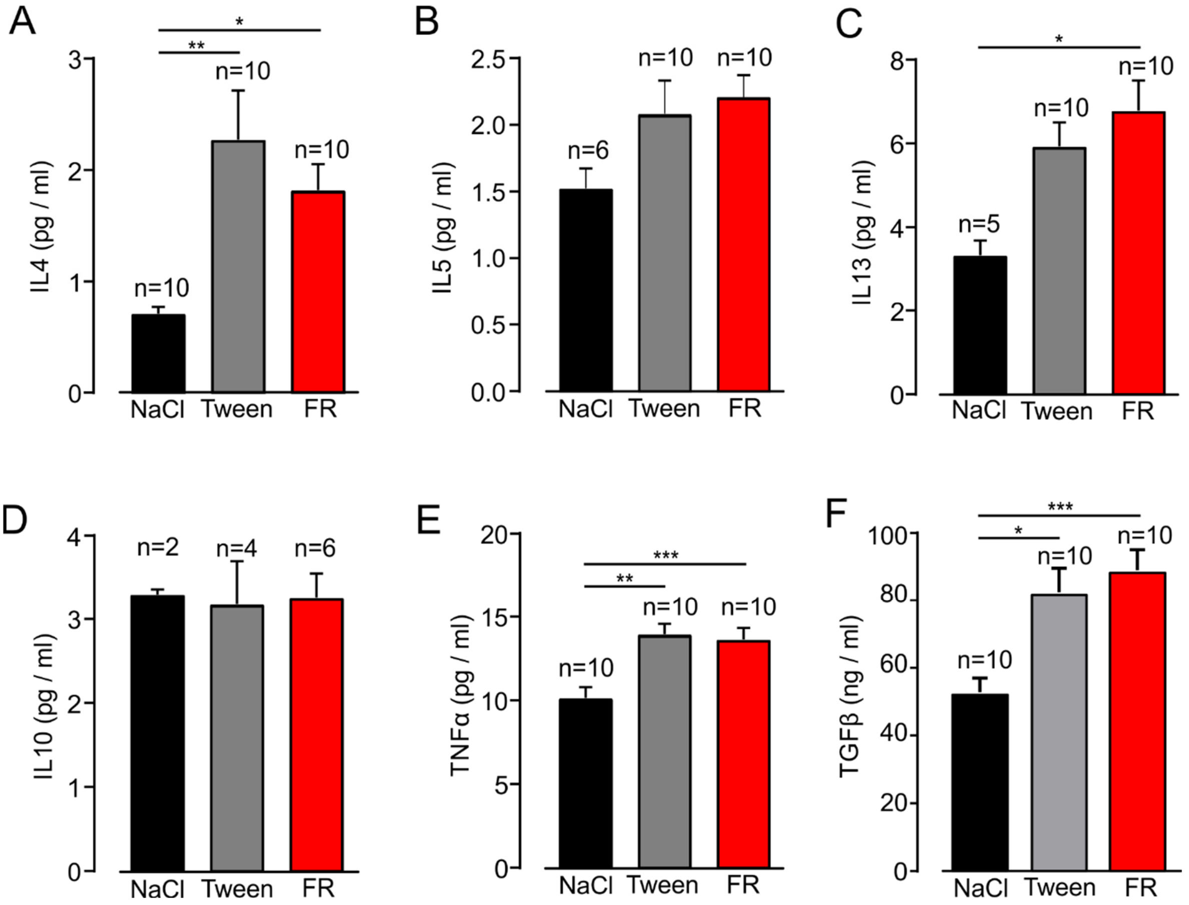

Figure S2. FR does not affect cytokine concentrations in the OVA-induced mouse model of chronic asthma. A-F) Cytokine levels of IL4 (A), IL5 (B), IL13 (C), IL10 (D), TNFα (E) and TGFβ (F) in lung homogenates of OVA-exposed animals after in vivo treatment with FR or the solvent compared to control animals, n represents the number of animals, *P<0.05, **P<0.01, ***P<0.001. A-F) One-way ANOVA, Tukey`s post hoc test.

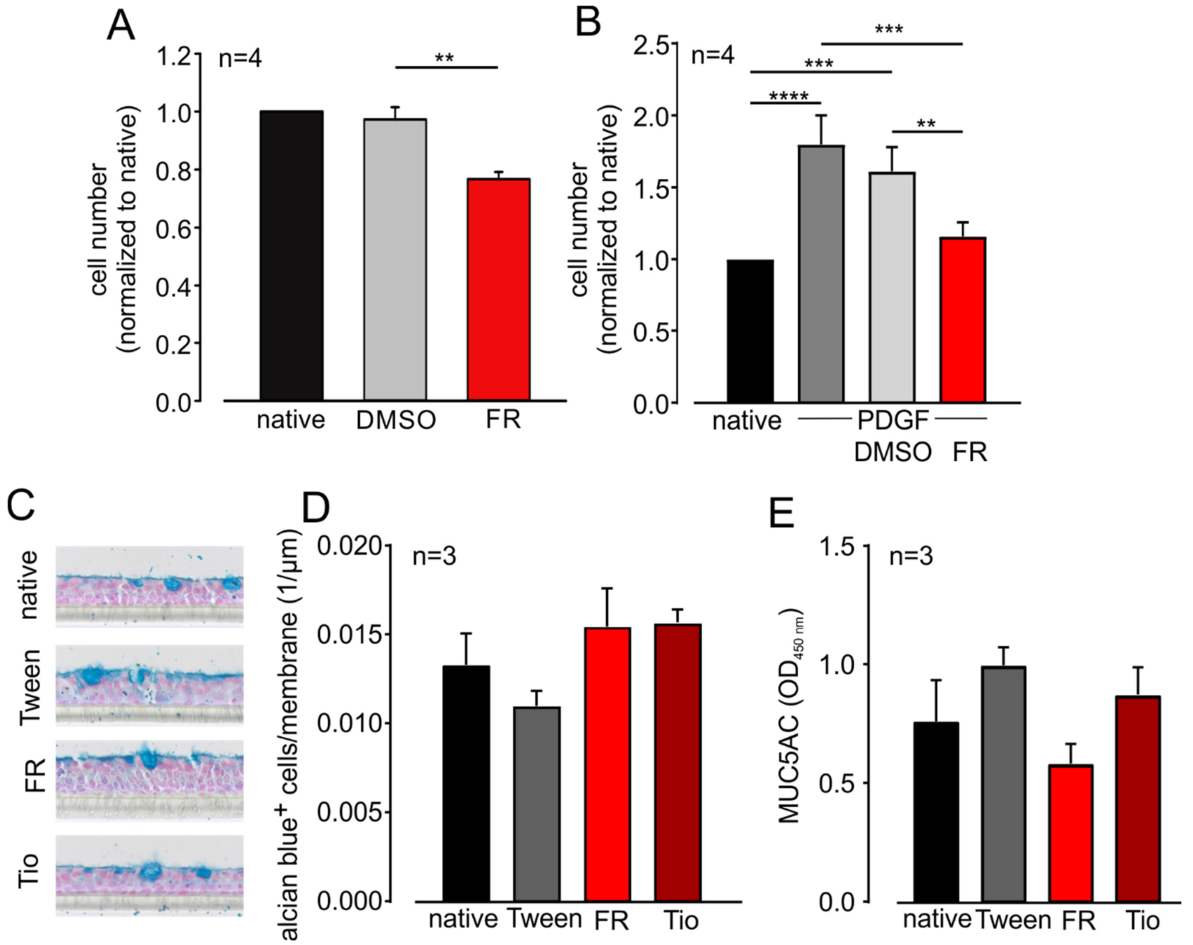

Figure S3. FR differentially modulates HFL1 cell growth and alcian blue+ goblet cell number of ALI cultures without pre-stimulation. A) Cell counts of HFL1 cells after treatment with FR or solvent. B) Cell counts of HFL1 cells after stimulation with PDGF BB (20 ng/ml) alone and treatment with FR (1 µM) or solvent DMSO for 5 days. C-E) Alcain blue staining of ALI cultures (C), number of alcian blue+ cells (D) and MUC5AC protein in the supernatant (E) after treatment with FR, tiotropium (Tio) or the solvent, n represents the number of different cell passages (A) or different wells (C,D), **P<0.01. A) One-way ANOVA, Tukey`s post hoc test.
