# Supplementary material for: Pharmacological Gq targeting prevents asthmatic airway remodeling
Source: Mol Ther. 2025 Jul 23;33(10):5149–61. doi: 10.1016/j.ymthe.2025.07.032 (PMC12848170; doi:10.1016/j.ymthe.2025.07.032)
Supplement: Document S2. Article plus supplemental information [file mmc2.pdf]

Molecular Therapy
Original Article

Pharmacological Gq targeting prevents asthmatic airway remodeling

Jennifer M. Dietrich,[2] Michaela Matthey,[1] Annika Simon,[1] Alexander Seidinger,[1] Cynthia Koziol-White,[3] Reynold A. Panettieri, Jr.,[3] Bernd K. Fleischmann,[2] and Daniela Wenzel[1,2]

[1]Institute of Physiology, Department of Systems Physiology, Medical Faculty, Ruhr University of Bochum, 44801 Bochum, Germany; [2]Institute of Physiology I, Life&Brain Center, Medical Faculty, University of Bonn, 53127 Bonn, Germany; [3]Rutgers Institute for Translational Medicine and Science, The State University of New Jersey, Rutgers, New Brunswick, NJ08901, USA

Airway remodeling is a critical hallmark of chronic asthma that is very difficult to treat. We have investigated this pathological process and found that local application of the pharmacological Gq inhibitor FR900359 (FR) attenuates the main features of airway remodeling, namely collagen deposition and goblet cell metaplasia in the chronic ovalbumin-induced asthma model in mice. We have explored the molecular mechanisms underlying the FR action and demonstrate that FR mitigates the growth of human lung fibroblasts in response to pathological stimuli *in vitro*. Likewise, FR inhibits mucus production and secretion in air-liquid interface cultures of human bronchial epithelial cells and in a human lung mucoepidermoid cell line. Notably, FR blocks mucus secretion in human lung slices from asthmatic patients. Thus, Gq proteins play a critical role in airway remodeling, hence pharmacological inhibition of Gq signaling represents a promising strategy for the treatment of chronic asthma.

INTRODUCTION

G protein-coupled receptors (GPCRs), which bind to Gq, are key regulators of lung physiology modulating smooth muscle tone and hence airway constriction.[1] Increased airway contractility, known as airway hyperresponsiveness (AHR), is a hallmark of asthma and is responsible for the typical asthma attacks, which can be life-threatening. In addition to AHR, the pathophysiology of asthma is often characterized by chronic inflammation in and around the airways, which is treated with glucocorticoids or monoclonal antibodies targeting cytokines.[2] However, over time such pharmacological options become less effective, as patients with asthma develop persistent airway constriction and airway remodeling. This is defined by a progressive pathological reorganization of the airways comprising airway wall thickening, fibroblast activation, extracellular matrix deposition, goblet cell metaplasia, and mucus hypersecretion, resulting in a further restriction of airflow.[3] In chronic asthma, these structural changes are known to induce persistent airway obstruction,[4] representing a major obstacle to effective therapies. Airway remodeling can occur through both pro-inflammatory signaling and mechanostimulation.[5,6] Targeting inflammation is currently a widely accepted strategy to prevent or reverse remodeling but a significant number of patients do not respond to anti-inflammatory drugs or show inconsistent responses.[7] In addition, there is evidence that airway remodeling may not necessarily be a consequence of inflammation, but acts independently.[8,9] Thus, new approaches that directly target airway remodeling are urgently required. Given that pharmacological inhibition of Gq proteins with the highly selective pan-Gq inhibitor FR900359 (FR) has been shown to be very effective in attenuating AHR[10] and AHR and remodeling have been discussed to be interdependent,[11,12] herein, we have examined the potential impact of Gq inhibition on airway remodeling in human cells and tissues as well as in a mouse model of chronic asthma *in vivo*.

We demonstrate that local application of FR to the lung strongly reduces AHR, collagen deposition, and goblet cell metaplasia in the mouse model of ovalbumin (OVA)-induced chronic asthma. Moreover, FR attenuates the growth of human lung fibroblasts, mucus production, and secretion in air-liquid interface (ALI) cultures of human bronchial epithelial cells or a human lung mucoepidermoid cell line. Most importantly, FR also prevents mucus release from human asthmatic lung sections. Thus, Gq inhibition may be a powerful direct approach to alleviate airway remodeling.

RESULTS

FR reduces airway remodeling in the chronic OVA model *in vivo*

First, we assessed the effect of the pan-Gq inhibitor FR on airway remodeling *in vivo*. To this aim, asthma was induced in Balb/c mice using the chronic OVA model. The development of asthma was proven by strong airway hyperresponsiveness to methacholine (MCh) in OVA animals as determined by flexiVent measurements (Figure 1A). We also detected, as expected, a marked increase in bronchoalveolar lavage (BAL) eosinophil counts in these animals, whereas macrophages were the predominant cell type in controls (Figures 1B and S1A). In mice treated with intratracheal application of FR (5 μg per mouse) prior to each OVA challenge, airway hyperresponsiveness was greatly diminished (Figure 1C) compared with the solvent. This proved the efficacy

Received 10 March 2025; accepted 18 July 2025;
https://doi.org/10.1016/j.ymthe.2025.07.032.

Correspondence: Daniela Wenzel, MD, Institute of Physiology, Department of Systems Physiology, Medical Faculty, Ruhr University of Bochum, 44801 Bochum, Germany.
E-mail: daniela.wenzel@rub.de

Published by Elsevier Inc. on behalf of The American Society of Gene and Cell Therapy.
This is an open access article under the CC BY license (http://creativecommons.org/licenses/by/4.0/).

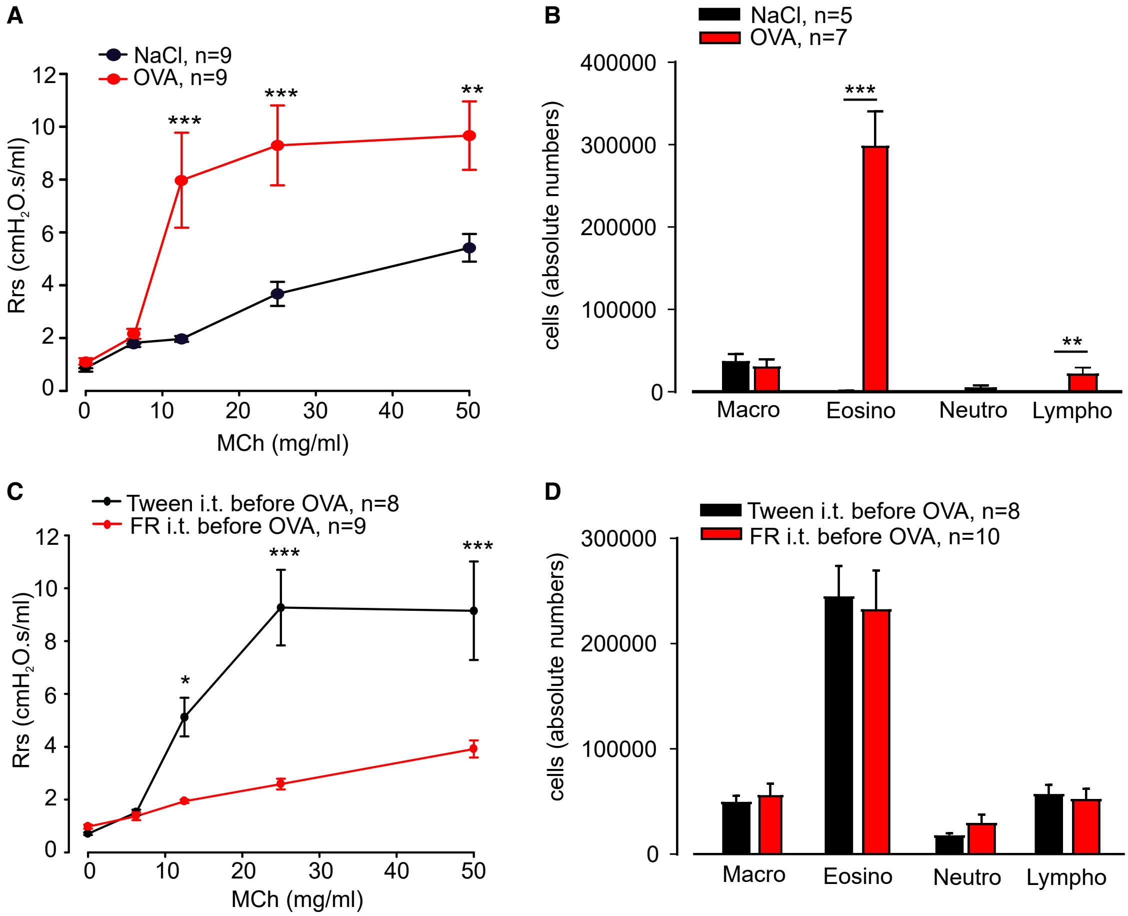

Figure 1. FR prevents airway hyperresponsiveness but not inflammation in the OVA-induced mouse model of chronic asthma

(A and B) Methacholine (MCh) dose-response curves for respiratory system resistance (A) and absolute cell counts (B) in BAL fluid for control and OVA-exposed Balb/c mice with chronic asthma. (C and D) MCh dose-response curves for respiratory system resistance (C) and absolute cell counts (D) in BAL fluid for FR- or solvent-treated OVA-sensitized Balb/c mice, n represents the number of animals, *$p < 0.05$, **$p < 0.01$, ***$p < 0.001$. (A) and (C) Two-way ANOVA, Bonferroni's multiple comparison test. (B) and (D) Unpaired two-tailed Student's t test.

of Gq inhibition on airway tone when applied repeatedly during chronic asthma development. Interestingly, BAL cell counts and eosinophil fractions remained unchanged by FR treatment (Figures 1D and S1B).

The lack of effect of FR on the inflammatory response was further supported by the finding that levels of cytokines and the transforming growth factor (TGF)β, which have been reported to play a role in asthma, were found to be unaltered in lung homogenates from FR-treated mice (Figure S2). We then analyzed airway remodeling in the mice and performed various stainings and morphometric analyses on paraffin-embedded lung sections. OVA induction enhanced collagen deposition in the peribronchial areas as assessed by Sirius red staining (Figures 2A–2C) and we found that this could be reduced by FR administration (Figures 2D–2F). In addition, we detected bronchial goblet cell metaplasia by periodic acid-Schiff (PAS) staining of mucus (Figures 2G–2L) and by specific staining for the glycoprotein MUC5AC (Figures 2M–2R). Quantification of both stainings revealed a high number of mucus-containing goblet cells in OVA-sensitized mice (Figures 2G–2I and 2M–2O), whereas no goblet cells were found in control animals. When we applied FR, this treatment strongly diminished the number of PAS+ and MUC5AC+ cells (Figures 2J–2L and 2P–2R), suggesting that FR reduced mucus production and/or the number of mucus-secreting cells.

We therefore examined epithelial cell differentiation and related pathways in FR-treated lungs by qPCR analysis of lung homogenates. Again, Muc5ac expression was strongly upregulated in OVA mice and could be diminished by FR administration (Figure 3A). The expression of the transcription factor SAM-pointed domain-containing ETS-like factor (Spdef), which is known to regulate goblet cell differentiation,[13,14] was upregulated in the lungs of OVA mice

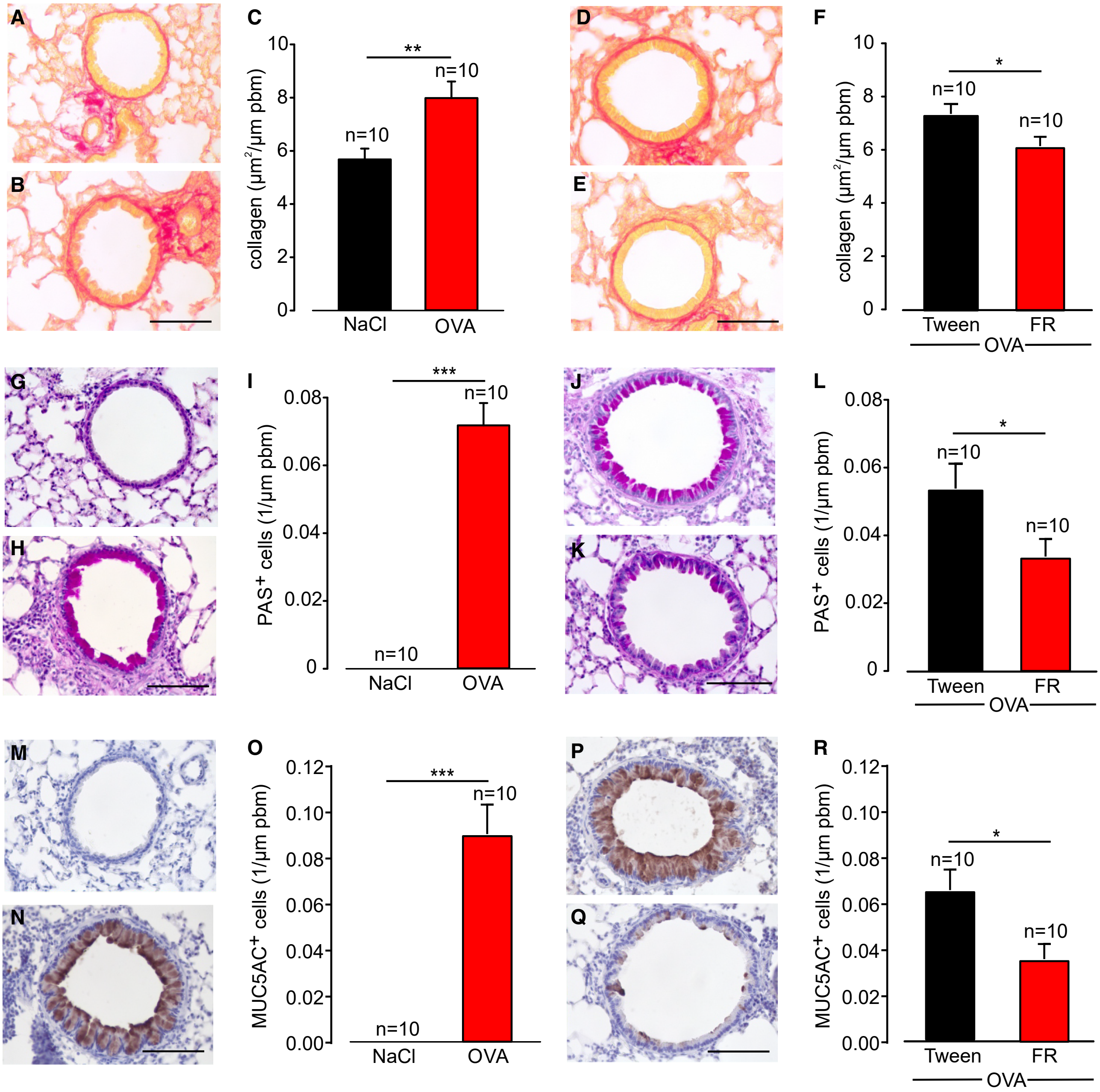

Figure 2. Local application of FR reduces collagen deposition and goblet cell metaplasia in the OVA-induced mouse model of chronic asthma

(A–F) Sirius red staining (A, B, D, and E) and quantification of collagen deposition (C and F) in control and OVA-exposed Balb/c mice (A–C) as well as FR (5 μg/mouse, intratracheal before each OVA challenge)- and solvent-treated OVA-sensitized Balb/c mice (D–F). (G–L) PAS staining (G, H, J, and K) and quantification of goblet cells (I and L) in control and OVA-exposed Balb/c mice (G–I) as well as FR- and solvent-treated OVA-sensitized Balb/c mice (J–L). (M–R) MUC5AC staining (M, N, P, and Q) and quantification of goblet cells (O and R) in control and OVA-exposed Balb/c mice (M–O) as well as FR- and solvent-treated OVA-sensitized Balb/c mice (P–R); n represents the number of animals, all scale bars, 50 μm, *$p < 0.05$, **$p < 0.01$, ***$p < 0.001$. (C), (F), (I), (L), (O), and (R) Unpaired two-tailed Student's t test.

but was not affected by FR treatment (Figure 3B). We also assessed the expression levels of Foxa2, an inhibitor of goblet cell differentiation,[15] and its potential target Scgb1a1 that are both anti-inflammatory regulators known to be downregulated in asthma.[16] Indeed, we detected downregulation of both genes in OVA mice, but again, FR did not alter the expression levels (Figures 3C and 3D). These findings indicate that FR does not affect the differentiation of goblet cells in the OVA model *in vivo*.

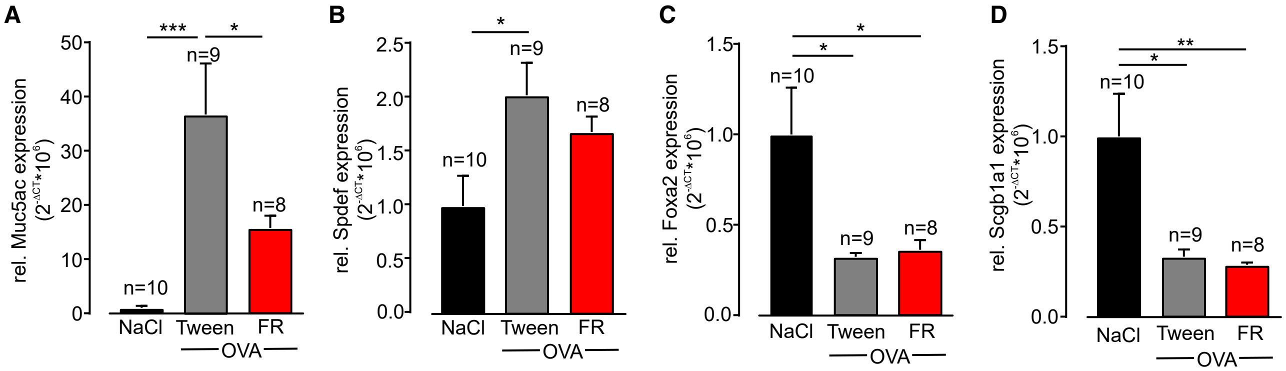

Figure 3. FR attenuates Muc5ac expression in the OVA-induced mouse model of chronic asthma

(A–D) Relative mRNA expression levels of Muc5ac (A), Spdef (B), Foxa2 (C), and Scgb1a1 (D) in lung homogenates of control animals or OVA-exposed Balb/c mice treated with FR or the solvent Tween; n represents the number of animals $^{*}p < 0.05$, $^{**}p < 0.01$, $^{***}p < 0.001$. (A)–(D) One-way ANOVA, Tukey's post hoc test.

FR inhibits cell growth of human lung fibroblasts, HFL1s

To analyze the underlying mechanisms of FR-dependent remodeling inhibition and to translate the findings to humans, we then performed *in vitro* experiments. As FR inhibited collagen deposition around the airways, and collagen is most likely produced by lung fibroblasts, we next assessed the effect of FR in the HFL1 cell line. Immunofluorescence staining revealed a pronounced expression of Gq proteins in this cell type (Figure 4A). Sirius red staining confirmed collagen accumulation within the cells (Figure 4B). First, we treated native HFL1s or cells stimulated with the asthma-related platelet-derived growth factor (PDGF) with FR (1 μM) or the solvent DMSO and found that FR reduced cell growth, whereas the solvent had no effect (Figures S3A and S3B). Then, we analyzed the effect of FR on the response of HFL1s to a combination of various asthma-related growth factors and Gq-dependent agonists, as such combinations are known to strongly induce airway remodeling processes.[17]

Our analysis revealed that a combination of PDGF (20 ng/mL, Figures 4B and 4C), epidermal growth factor (EGF) (60 ng/mL, Figure 4D), and TGFβ (5 ng/mL, Figures 4E–4G) with thrombin (1 U/mL, Figures 4B–4E), ATPγS (100 μM, Figure 4F), or carbachol (CCh) (100 μM, Figure 4G) strongly stimulated cell growth after 5 days. FR diminished HFL1 cell growth to almost the level of native cells without growth factor treatment, while the solvent DMSO had no effect. Thus, FR reduces the growth of collagen-producing fibroblasts in response to asthma-related growth factors.

FR prevents mucus secretion of goblet cells in an ALI culture

To further investigate the effects of FR on goblet cell function and mucus production *in vitro*, we used air-liquid interface (ALI) cultures with normal human bronchial epithelial cells (NHBECs). In this model, airway epithelial cells are differentiated into a pseudostratified epithelium exhibiting morphological and functional characteristics similar to human airway epithelium. First, we differentiated the cells without cytokine stimulation and applied FR, the solvent Tween, or the muscarinic M3 receptor antagonist tiotropium (Tio, 10 nM) during differentiation. Three weeks after the airlift, we analyzed the cultures and labeled mucus in goblet cells with Alcian blue staining (Figure S3C). In addition, we assessed MUC5AC secretion in the supernatant that was collected during weeks 2 and 3 of differentiation. Counting of Alcian blue+ cells (Figure S3D) and analysis of MUC5AC protein (Figure S3E) revealed no effect of FR or Tio on the number of goblet cells and mucus secretion without pre-stimulation. Then, we stimulated the cells with a combination of the cytokine interleukin (IL)-13 (1 ng/mL) with or without asthma-related Gq-dependent agonists to enhance goblet cell differentiation. Some of the wells were incubated with FR, the solvent Tween, or Tio. As expected, IL-13 increased the number of Alcian blue+ goblet cells during differentiation (Figures 5A, 5C, and 5E). Interestingly, additional treatment with the Gq-dependent agonists CCh (100 μM), ATPγS (100 μM), and thrombin (1 U/mL) resulted in a reduced number of mucus-containing goblet cells as compared with IL-13 alone. FR prevented this effect for all Gq-dependent agonists, whereas Tio was only effective for the M3 agonist CCh (Figures 5A, 5C, and 5E). Analysis of mucus secretion in the supernatant revealed high levels of MUC5AC after stimulation with Gq-dependent agonists and reduced levels after FR treatment regardless of the type of Gq-dependent agonist; again, Tio was effective only after CCh stimulation (Figures 5B, 5D, and 5F). These data suggest that Gq-dependent agonists stimulate mucus secretion during goblet cell differentiation, resulting in high MUC5AC concentrations in the supernatant. As a result, fewer goblet cells contain mucus and can be detected by Alcian blue staining. Thus, the treatment of bronchial epithelial cells in ALI cultures with Gq-dependent agonists + IL-13 during differentiation may affect both mucus production and mucus secretion, making it impossible to distinguish between these two processes in this setting.

To prove that FR can prevent mucus secretion by Gq-dependent agonists, we next treated ALI cultures with Gq-dependent agonists and FR or Tio after differentiation had been accomplished. Differentiation of the cells in the presence of IL-13 induced a strong increase of Alcian blue+ goblet cells after 3 weeks (Figures 6A, 6B, 6D, 6E, 6G, and 6H). After the removal of residual mucus on the cell surface

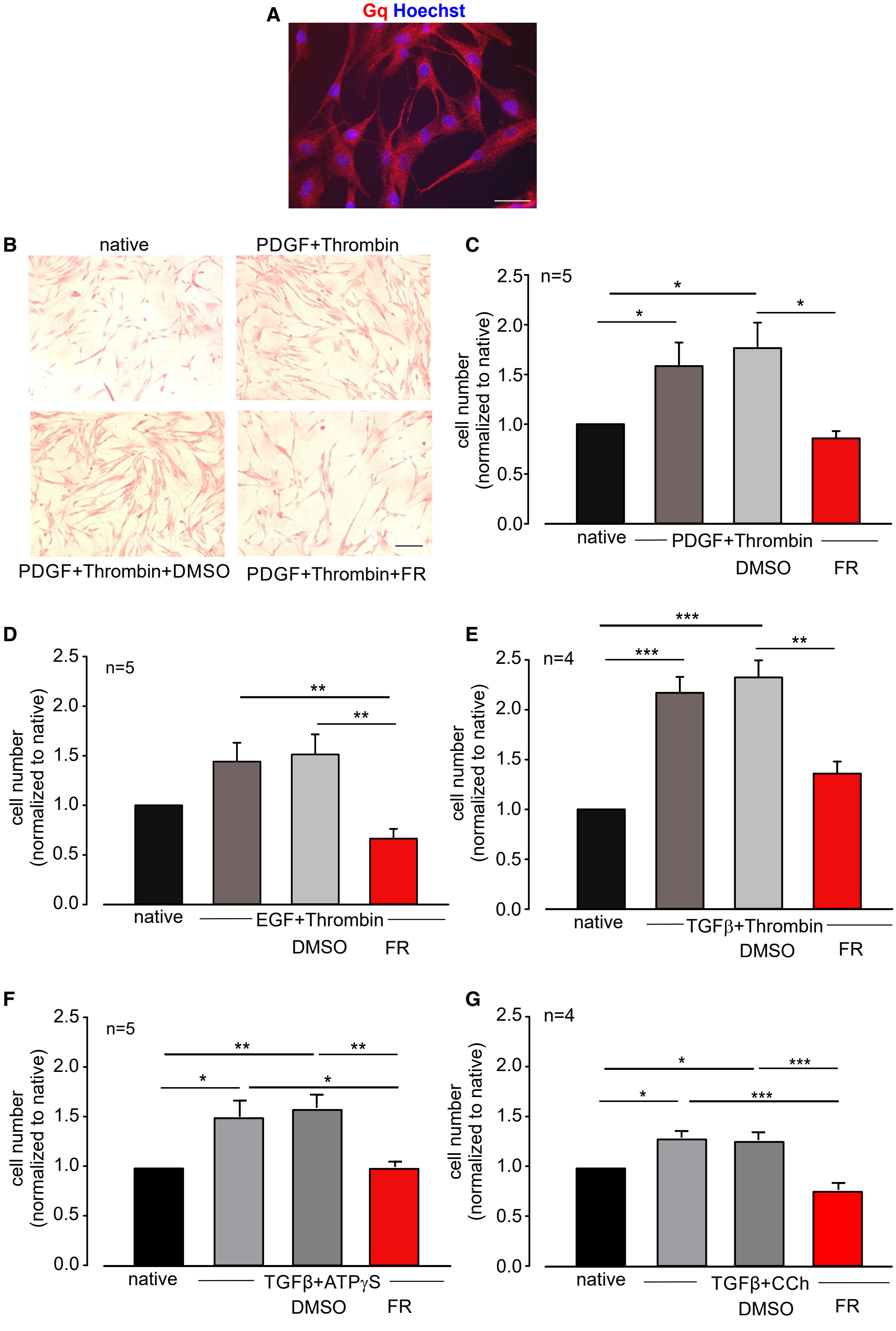

(legend on next page)

layer by a washing step, cells were preincubated with the solvent Tween, with FR, or with Tio for 30 min. Then, mucus secretion was acutely stimulated for another 30 min with the Gq-dependent agonists CCh (Figures 6A–6C), ATP (Figures 6D–6F), or thrombin (Figures 6G–6I). The number of mucus-filled goblet cells was analyzed by counting of Alcian blue+ cells, and mucus secretion in the supernatant was assayed by ELISA. These experiments demonstrate that acute application of the Gq-dependent agonists decreased numbers of Alcian blue+ goblet cells (Figures 6A, 6B, 6D, 6E, 6G, and 6H) and elevated levels of MUC5AC in the supernatant (Figures 6C–6F and 6I). FR could prevent both effects, whereas the M3 antagonist Tio only inhibited the effects in response to CCh treatment. Thus, mucus secretion by Gq-dependent agonists is inhibited by FR.

FR prevents mucus production in NCI-H292 cells and mucus secretion in human lung slices from asthmatic patients

To assess whether FR can prevent not only mucus secretion but also mucus production, we used the human pulmonary mucoepidermoid cell line NCI-H292. In this cell line, similar to human asthmatic airways, the EGF receptor is expressed and its stimulation typically results in upregulation of MUC5AC and mucus production.[18] First, we performed immunostaining of NCI-H292 cells and found that they strongly express Gq proteins (Figure 7A). Then, we stimulated the cells with a combination of EGF (60 ng/mL) and the Gq-dependent agonist thrombin (1 U/mL). Thrombin was previously identified in the sputum of asthmatic patients,[19] and together with EGF, it increased MUC5AC production in NCI-H292 cells. In some experiments, cells were additionally incubated with FR (0.5 μM) or the solvent Tween. After 4 days, qPCR for MUC5AC was performed and revealed that FR attenuated MUC5AC expression in this human cell line (Figure 7B). Next, we wanted to know if FR is also effective in human airways of asthma patients. Therefore, we generated precision-cut lung slices (PCLSs) derived[20] from the lungs of asthma patients. The slices were treated with FR (1 μM) or the solvent Tween for 72 h, and the MUC5AC concentration in the supernatant was determined by ELISA measurements. Our data reveal that FR, but not the solvent, strongly reduced mucus secretion in human asthmatic lung sections (Figure 7C), illustrating that FR is also effective in humans and even in asthmatic tissue.

DISCUSSION

The correlation of airway remodeling and persistent airway obstruction in asthma remains an enigma and challenges current therapeutic approaches.[21,22] Here, we show that the pharmacological Gq inhibitor FR reduces collagen deposition and goblet cell metaplasia in airways. We can further demonstrate that Gq-dependent agonists contribute to the proliferation of lung fibroblasts and to mucus production/secretion, making pharmacological Gq inhibition a promising treatment approach.

Because pharmacological Gq inhibitors have only recently become available, in the past the impact of Gq signaling on asthma development could be investigated only in genetic models.

A common finding of asthma development in animals treated with the pharmacological pan-Gq inhibitor FR or Gq−/−[23] and M3−/− mice[24] is the lack of AHR. This confirms the well-known important role of Gq and M3 for airway tone regulation in asthma pathophysiology.[25] However, in contrast to the results in Gq−/− mice, FR does not affect the number of inflammatory cells or cytokines in asthma. This may be explained by local inhibition of all Gq family members (Gq, G11, and G14) after pulmonary FR administration, whereas only Gq is missing in all tissues in Gq−/− animals. In fact, the absence of Gq in bone marrow cells was identified to be responsible for reduced eosinophilia in this mouse line.[26] In accordance with our results in FR-treated animals, M3−/− mice demonstrated no difference in pulmonary eosinophil counts in asthma compared with wild-type animals but also showed reduced signs of airway remodeling.[24,27] Effects of Gq activation on airway remodeling are consistent with earlier reports revealing that Gq activation does not only result in short-term effects but also plays a crucial role in regulating cell growth, migration, and differentiation in various tissues.[28–31] Also in pulmonary cells, GPCR/Gq stimulation has been reported to induce long-term remodeling effects, and specific underlying signaling molecules have been identified. For example, thrombin, histamine, carbachol, and M3 receptor activation stimulated airway smooth muscle growth synergistically with receptor tyrosine kinase activation. This effect was mediated via PKC-dependent glycogen synthase kinase-3 (GSK-3) inhibition,[32] Akt phosphorylation,[17] or P70 S6 kinase activation.[33] M3 receptor stimulation also increased MUC5AC expression in human airway epithelial cells via EGF receptor transactivation.[34] Thrombin, which is known to be elevated in asthma of humans and animals,[19,35] induced proliferation of lung fibroblasts by protease-activated receptor 1 (PAR1).[36]

In accordance with these results, our data reveal that FR strongly decreased collagen deposition *in vivo* and reduced human lung fibroblast growth *in vitro* after stimulation with a combination of disease-relevant growth factors and Gq-dependent GPCRs, which demonstrated synergistic effects on airway remodeling before.[32,37] Interestingly, *in vitro*, FR also inhibited growth of unstimulated fibroblasts or cells exclusively stimulated by receptor tyrosine kinases suggesting that Gq shows basal activity in these cells. Thus, Gq

Figure 4. FR inhibits the growth of human lung fibroblasts (HFL1s) in culture
(A) Immunostaining of Gq in HFL1 cells, red: Gq, blue: Hoechst, scale bar, 50 μm. (B) Sirius red staining of HFL1 cells stimulated with PDGF + thrombin and treated with FR or solvent; scale bar, 200 μm. (C–E) Cell counts of HFL1 cells after stimulation with PDGF (20 ng/mL, C), EGF (60 ng/mL, D), or TGFβ (5 ng/mL, E) + thrombin (1 U/mL) and treatment with FR (1 μM) or solvent DMSO for 5 days. (F and G) Cell counts of HFL1 cells after stimulation with TGFβ + ATPγS (100 μM, F) or CCh (100 μM, G) and treatment with FR (1 μM) or solvent DMSO for 5 days; n represents the number of different cell passages, *$p < 0.05$, **$p < 0.01$, ***$p < 0.001$. (C)–(G) One-way ANOVA, Tukey's post hoc test.

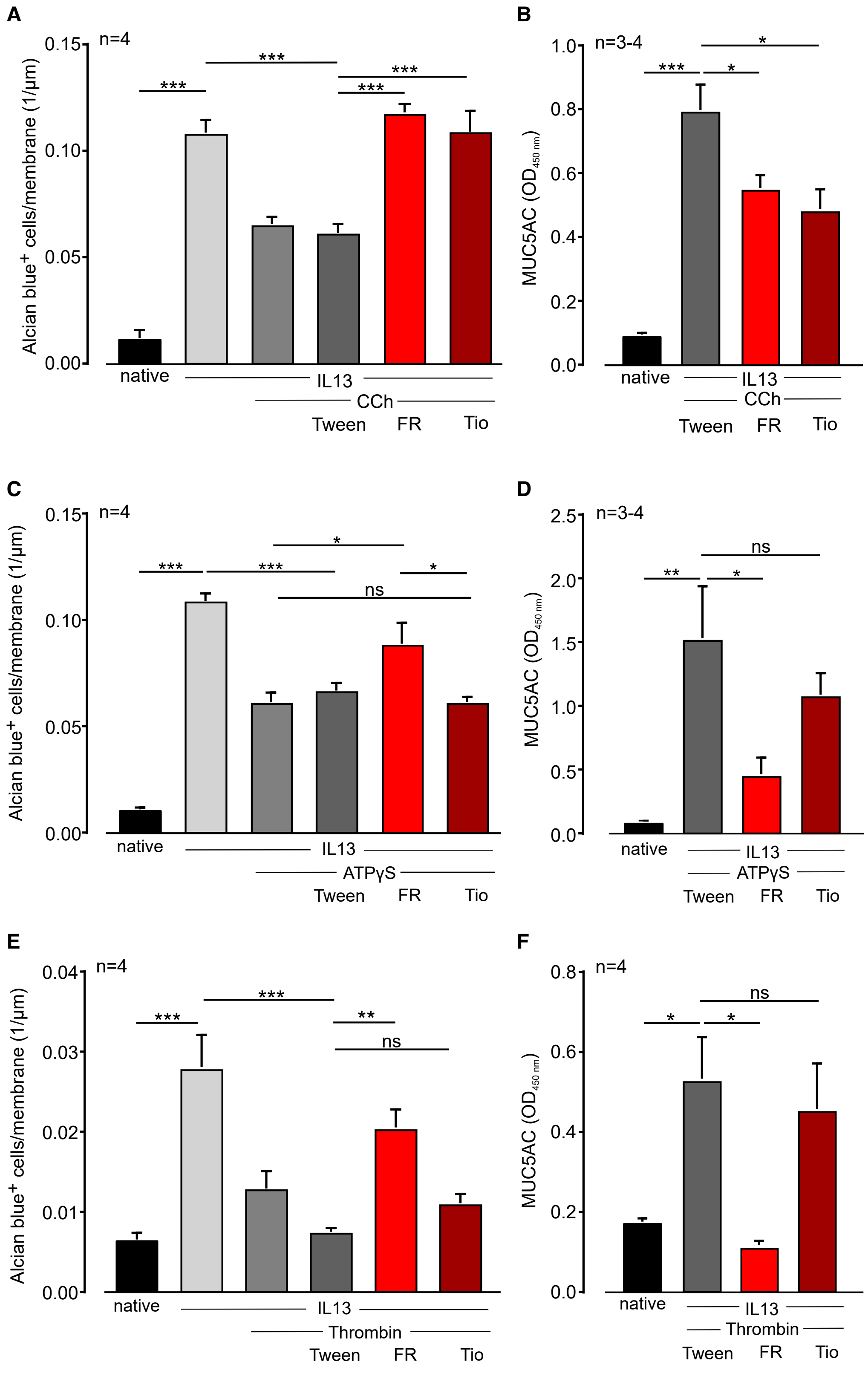

(legend on next page)

proteins are important targets to prevent fibroblast growth and collagen deposition. Besides the reduced collagen deposition in the lungs of FR-treated asthmatic mice, we found a reduction in PAS+ and MUC5AC+ goblet cells in histological lung sections as well as reduced Muc5ac expression in lung homogenates. However, there were no differences in the pulmonary mRNA expression of transcription factors regulating goblet cell differentiation. Therefore, we hypothesized that FR does not influence the differentiation or quantity of these cells but rather affects the mucus production and secretion of the goblet cells. To further investigate this, we employed a mucoepidermoid cell line and ALI cultures in combination with established stimulation protocols known to induce goblet cell metaplasia and mucus production/secretion by asthma-related stimuli.[18,34,38] In the mucoepidermoid cell line, the reduced MUC5AC expression in response to FR indicates that FR inhibits mucus production. In addition, in ALI cultures with acute stimulation of mucus secretion by CCh, ATP, or thrombin, FR increased the number of MUC5AC-containing goblet cells and decreased MUC5AC levels in the supernatant of the airway epithelium. This clearly points toward the inhibition of mucus secretion by FR, which is supported by attenuated mucus secretion in lung slices from human asthma patients. Thus, our data suggest that mucus production and secretion in response to asthma-related stimuli are Gq-dependent and can be inhibited by FR.

The different effects of FR on the number of goblet cells in OVA-sensitized mice and ALI cultures may be explained by the differences in these experimental models. Under *in vivo* conditions, asthma pathophysiology is affected by various cytokines, different cell types, and airway constriction, all absent in ALI cultures. Our results suggest that FR inhibits AHR and airway remodeling downstream of inflammation. Whether FR directly affects both *in vivo* remains to be determined, as a mutual dependency between AHR and remodeling is discussed,[12,39] which may account for a part of the FR effect. Moreover, it cannot be excluded that changes in AHR depend, at least in part, on alterations in the pulmonary vasculature,[40] which is also a target of FR.[41]

Because there are currently no drugs specifically targeting airway remodeling, pan-Gq inhibition could fill this gap and be of therapeutic relevance. For the *in vivo* experiments, we selected a dosage of 5 μg FR per mouse per day prior to each OVA challenge. This treatment regimen was chosen based on its proven effectiveness and lack of obvious side effects in our previous study on the role of FR in an animal model of acute asthma.[10] Given that Gq proteins are also present in other organs, this lack of side effects may be attributed to the localized intratracheal application of the compound to the lung. In fact, our earlier research using a newly developed radioligand for FR demonstrated the accumulation of FR in the lung following intratracheal application.[42] However, we cannot entirely rule out a potential impact on other Gq-dependent processes, such as blood pressure regulation or platelet aggregation, with chronic FR application over extended periods.

For *ex vivo*/*in vitro* experiments, we have chosen FR concentrations ranging from 0.5 to 10 μM to avoid any solvent-related effects depending on the assay. These dosages proved to inhibit Gq-dependent effects almost completely in our previous experiments[10] and demonstrated excellent specificity.[28,43,44] An important advantage of FR treatment is that it is a pan-Gq inhibitor therefore inhibiting multiple GPCR-dependent pathways signaling via Gq. Thus, FR inhibits a central signaling hub for airway regulation, which not only regulates AHR but also airway remodeling in asthma, involving fibroblast growth and collagen deposition as well as mucus production and secretion. The high level of efficacy is underscored by our very promising experimental results obtained in human lung sections from asthma patients. Further experiments and analyses in asthmatic tissues and disease models are required to determine the full potential of FR as a bonafide modulator of airway remodeling.

MATERIALS AND METHODS

Animal experiments

Eight- to 10-week-old female Balb/c mice (Charles River Laboratories) were used. Animal housing and experiments were approved by the local ethics committee and conducted according to the guidelines of the German law of protection of animal life with approval by the local government authorities (Landesamt für Natur, Umwelt und Verbraucherschutz Nordrhein-Westfalen (LANUV), NRW, Germany).

Chronic OVA-induced asthma model

Mice were sensitized with intraperitoneal injections of 20 μg OVA (Sigma-Aldrich, Germany) and 2 mg Alum (Thermo Scientific, USA) dissolved in PBS (100 μL, intraperitoneally [i.p.]) on days 0 and 14. For the following 3 weeks mice were exposed to 1% OVA (5 mL) or NaCl (control) as an aerosol for 20 min by inhalation on days 21–23, 28–30 and 35–37. FR (5 μg per mouse) dissolved in 50 μL Tween 0.1% or the solvent alone was applied intratracheally one hour before each OVA challenge according to the procedure previously described.[41] Analysis was performed on days 38 and 39.

Generation of human PCLSs from asthma patients

The human tissue was provided by the National Disease Research Interchange (NDRI) and the International Institute for the Advancement of Medicine (IIAM) in a de-identified manner. The Rutgers

Figure 5. FR increases the number of Alcian blue+ goblet cells in ALI cultures of human bronchial epithelial cells

(A and B) Number of Alcian blue+ cells in ALI culture (A) and MUC5AC protein in the supernatant (B) after stimulation with IL-13 (1 ng/mL) and CCh (100 μM) and additional treatment with FR (100 μM), tiotropium (10 nM, Tio), or the solvent Tween. (C and D) Number of Alcian blue+ cells in ALI culture (C) and MUC5AC protein in the supernatant (D) after stimulation with IL-13 and ATPγS (100 μM) and additional treatment with FR, Tio, or the solvent Tween. (E and F) Number of Alcian blue+ cells in ALI culture (E) and MUC5AC protein in the supernatant (F) after stimulation with IL-13 and thrombin (1 U/mL) and additional treatment with FR, Tio, or the solvent Tween; n represents the number of different wells, *$p < 0.05$, **$p < 0.01$, ***$p < 0.001$. (A)–(F) One-way ANOVA, Tukey's post hoc test.

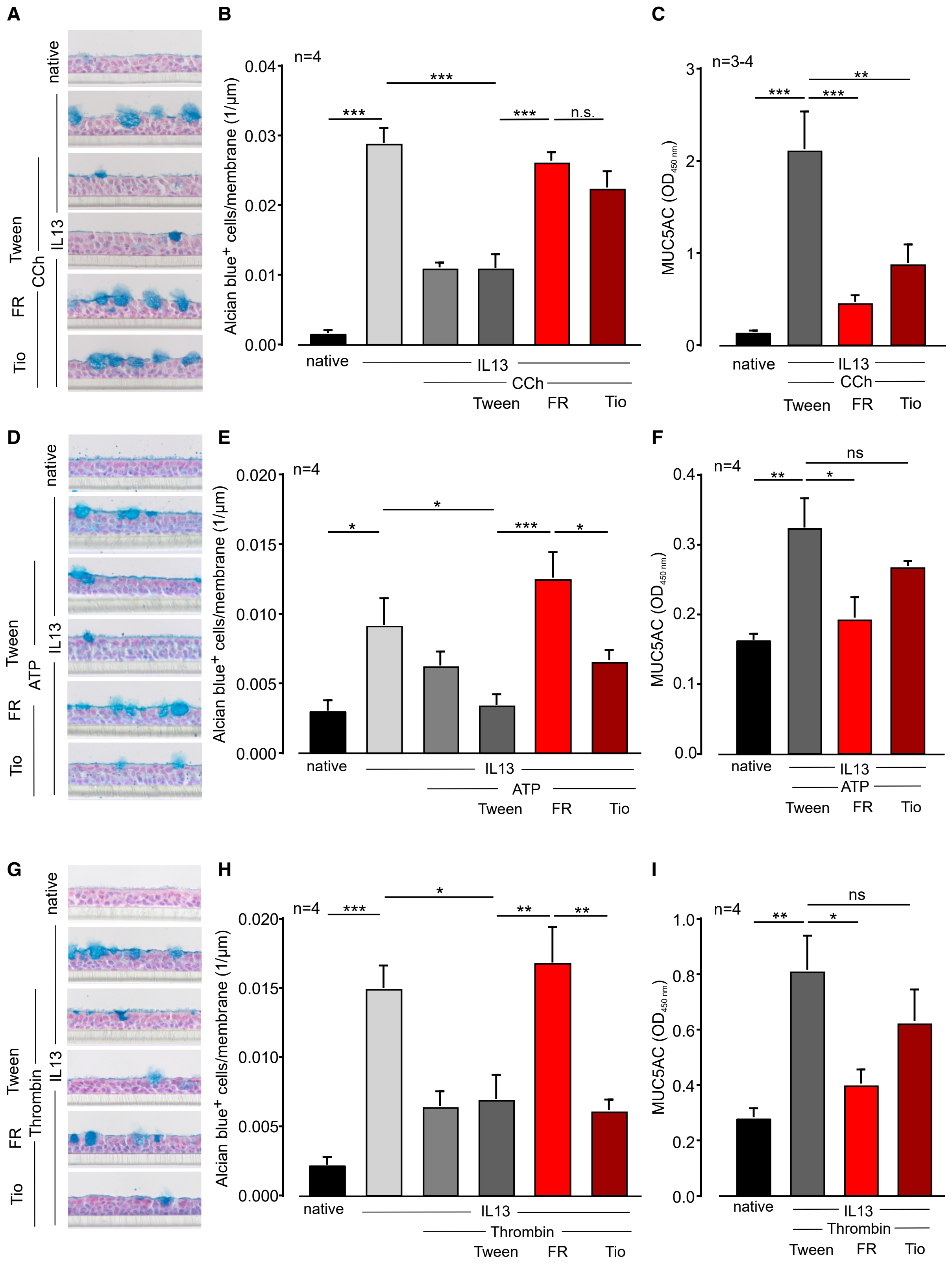

(legend on next page)

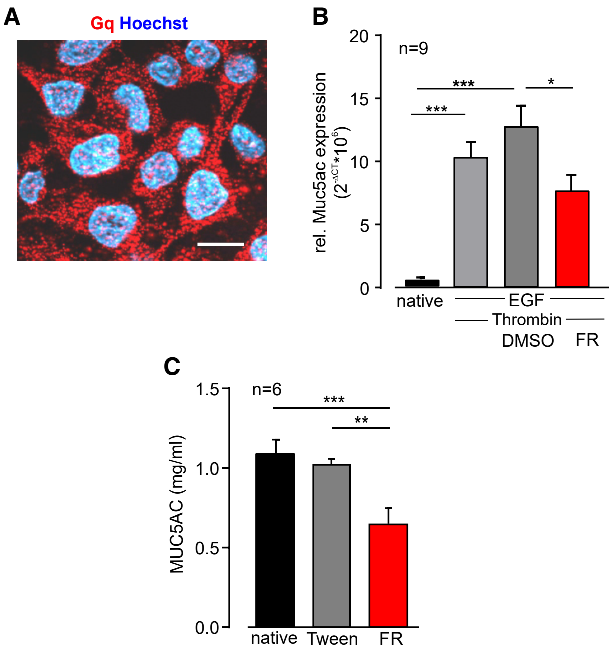

Figure 7. FR limits MUC5AC expression in human lung mucoepidermoid cell line NCI-H292 and attenuates mucus secretion in human lung slices of asthmatic patients

(A) Immunostaining of Gq in NCI-292 cells, red: Gq, blue: Hoechst, scale bar, 10 μm. (B) Relative Muc5ac mRNA expression in NCI-H292 cells after stimulation with EGF (60 ng/mL) and thrombin (1 U/mL) and treatment with FR (0.5 μM) or the solvent for 4 days. (C) MUC5AC protein in the supernatant of human lung slices from asthma patients after treatment with FR (1 μM) or the solvent; n represents the number of different cell passages (B) or different human lung slices from one patient (C), *$p < 0.05$, **$p < 0.01$, ***$p < 0.001$. (C) and (D) One-way ANOVA, Tukey's post hoc test.

Institutional Review Board deemed this use of tissue as non-human subjects research.

PCLS preparation has recently been described.[45] Briefly, a lung lobe from an asthmatic donor was inflated with 2% (wt/vol) low melting point agarose. After solidification of the agarose, cores of 8-mm diameter with a central airway were generated and sectioned with a vibratome (thickness 350 μm). PCLSs were incubated with 1 μM FR in 200 μL Ham's F-12 medium at 37°C, 5% CO_2. After 72 h, the supernatant was collected and MUC5AC levels were determined by ELISA.

Cell culture

Human lung fibroblasts (HFL1s) were obtained from ATCC (Manassas, USA) and cultivated in DMEM supplemented with 10% fetal calf serum (FCS), 0.1 mM non-essential amino acids, 0.1 mM β-mercaptoethanol, and 1% penicillin/streptomycin (Gibco). Cells were passaged at 80% confluence and used up to passage 12. The lung mucoepidermoid NCI-H292 cell line was obtained from ATCC (CRL-1848) and cultivated in 10% RPMI supplemented with 1% penicillin/streptomycin.

Cell growth experiments using HFL1s

For cell growth experiments, 20,000 HFL1s were seeded per well in six-well plates and allowed to attach for 24 h in 10% DMEM. Then, they were starved for 48 h in 0.1% DMEM. After that, cell growth stimulators EGF (60 ng/mL, Sigma/Merck, Germany), TGFβ (5 ng/mL, Peprotech, USA), thrombin (1 U/mL, Enzo, Germany), and PDGF (20 ng/mL, Peprotech, USA) were added to 1% DMEM and distributed in the wells. FR (1 μM) or the solvent DMSO was added to each well. Cells were treated for 5 days and medium was changed daily. Twenty-four hours after the last treatment, cells from two wells were pooled and cell numbers were counted by using a Neubauer hemocytometer.

Stimulation of mucus production in NCI-H292 cells

NCI-H292 cells were starved for 48 h and then stimulated with EGF (60 ng/mL) and thrombin (1 U/mL) in 1% RPMI for 4 days. The medium was changed daily. Then, cells were lysed with TRIzol and RNA was isolated for qPCR experiments.

ALI culture

Human bronchial epithelial cells (HBECs) (CC-2540S, Lonza, Switzerland) were differentiated in transwell plates using ALI conditions (Stemcell Technologies, Canada). Briefly, 8.3×10^4 cells were seeded per insert and cultured in ExPlus basal medium (Stemcell Technologies) for about 5 days to confluence. Then, airlift was performed and ALI maintenance medium (Stemcell Technologies) supplemented with IL-13 (1 ng/mL, Peprotech, USA) was added to the lower compartments. Carbachol (CCh) (100 μM, Sigma/Merck), thrombin (1 U/mL), ATPγS (100 μM, Sigma/Merck), FR (10 μM), and tiotropium bromide (Tio) (10 nM) or the solvent Tween 80 were added to the lower and upper compartments. The medium was changed three times per week and the cultivation was continued for another 3 weeks. During the last 2 weeks of cultivation, mucus was collected by washing the call layer with 100 μL PBS and stored at −80°C for ELISA measurements. After differentiation, cells were fixated with Roti-Histofix 4% (Carl Roth, Germany). To study mucus secretion, differentiation was performed with IL-13 alone. After 3 weeks, the cells were starved for 48 h in ALI maintenance medium without supplements and the cells were washed six times with starving medium for 1 hour each time. Then, cells were pre-treated

Figure 6. FR prevents mucus secretion in differentiated ALI cultures of human bronchial epithelial cells

(A–C) Alcian blue staining of ALI cultures (A), number of Alcian blue+ cells (B), and MUC5AC protein in the supernatant (C) after stimulation with IL-13 (1 ng/mL) and CCh (100 μM) and additional treatment with FR (100 μM), Tio (10 nM), or the solvent Tween. (D–F) Alcian blue staining of ALI cultures (D), number of Alcian blue+ cells (E), and MUC5AC protein in the supernatant (F) after stimulation with IL-13 and ATP (100 μM) and additional treatment with FR, Tio, or the solvent Tween. (G–I) Alcian blue staining of ALI cultures (G), number of Alcian blue+ cells (H), and MUC5AC protein in the supernatant (I) after stimulation with IL-13 and thrombin (1 U/mL) and additional treatment with FR, Tio, or the solvent Tween; n represents the number of different wells, *$p < 0.05$, **$p < 0.01$, ***$p < 0.001$. (A)–(I) One-way ANOVA, Tukey's post hoc test.

with FR, Tio, or Tween for 30 min and mucus secretion was induced by CCh, thrombin, or ATP (100 μM, Sigma/Merck) for another 30 min. After that, the supernatant was collected for ELISA and the cells were fixated for histologic analysis.

qPCR analysis

qPCR was exerted as previously described.[46] Lung lobes were stored in RNA later (Thermo Fisher Scientific) at −80°C, and then homogenized in TRIzol using the TissueLyzer LT (Qiagen, Germany). RNA extraction was performed using TRIzol reagent. RNA concentrations were determined using the NanoDrop 100 Spectrometer (PeqLab, Germany). RNA integrity numbers (RINs) were assessed using the Bioanalyzer 2100 (Agilent, USA), only samples with RIN numbers above 7 were processed further. For cDNA transcription, the SuperScript VILO cDNA Synthesis Kit was applied (Invitrogen, USA). qPCR was performed with RotorGene SYBR Green PCR kits (Qiagen, Germany) using the following Quantitect Primer Assays (Qiagen, Germany): human MUC5AC (QT01329615), mouse Muc5ac (QT01196006), mouse Spdef (QT00107191), mouse Foxa2 (QT00242809), and mouse scgb1a1 (QT00105266). Relative gene expression was quantified by the $2^{\Delta CT}$-method using 18SrRNA as a housekeeping gene (QT01036875).

flexiVent measurements

flexiVent measurements were performed as described before.[10,47] Briefly, mice were anesthetized with fentanyl (50 μg/kg), medetomidine (0.5 mg/kg), and midazolam (5 mg/kg) i.p. on a heating plate (37°C). The trachea was exposed and cannulated and animals were ventilated with a tidal volume of 10 mL/kg at 150 breaths/min and a positive end-expiratory pressure of 2.5 cmH_2O. For optimal skeletal muscle relaxation, vecuronium was applied (0.9 mg/kg i.p.). Airway resistance was assessed with snapshot perturbations of the flexiVent (Scireq). To induce airway constriction, increasing doses of methacholine (MCh) (1, 6, 12.5, 25, and 50 mg/mL, 25 μL) were applied via the Aeroneb Lab nebulizer (AG-ALI1100, Aerogen).

Bronchoalveolar lavage, cell counts

After flexiVent measurements mice were euthanized. BAL fluid was collected by instillation of 1 mL ice-cold PBS containing 2 mM EDTA for three times via the trachea. BAL fluid was centrifuged at 600 rpm for 5 min at 4°C. Cells were resuspended in 1 mL PBS and counted. For staining, 200,000 cells were centrifuged onto slides using a cytospin centrifuge (Tharmac, Waldsolms, Germany). After drying, the cells were fixated and stained with DiffQuick reagent (Morphisto, Frankfurt a.M., Germany) according to the manufacturer's instruction. Ten images were captured by an Axiostar microscope (Zeiss, Germany) and cell counts were determined based on morphologic criteria.

ELISA measurements

For MUC5AC ELISA measurements, supernatants of ALI cultures were diluted (1:200–1:400) in PBS and 50 μL was applied as duplicates to individual wells of a 96-well plate (Falcon, Corning, USA). After shaking, the lid of the plate was removed and the samples were dried under a cell culture bench overnight. The next day, the plate was washed three times and blocked with 1% bovine serum albumin (BSA) (Sigma/Merck) for 20 min. Then, the plate was incubated with biotinylated MUC5AC antibody in 0.1% BSA (1:500, MAS-12175, Invitrogen, USA) for 2 h at room temperature. After washing, horseradish peroxidase-conjugated streptavidin (1:5,000, Thermo Fisher Scientific, USA) was incubated for 30 min and 100 μL of tetramethylbenzidine (TMB) substrate solution (Invitrogen) was added and stopped with 1 M HCl at visual control. Absorbance was measured at 450 nm using a Tecan Spark Reader (Tecan, Switzerland).

For TGFβ ELISA measurements, frozen lung tissue was homogenized in 500 μL RIPA buffer and protein concentration was assessed using the Pierce BCA Protein Assay Kit (23225, Thermo Scientific, USA). TGFβ levels were measured using the mouse TGFbeta 1 ELISA Kit (Ab119557, Abcam, USA) according to manufacturer's instructions.

Histological staining and immunostaining

For the staining of lung sections, lungs were inflation-fixated using Roti-Histofix 4%. Then, paraffin sections of 5-μm thickness were generated and stained with Sirius red as well as periodic acid-Schiff (PAS). For MUC5AC staining, antigen retrieval was performed (15 min, 93°C, citrate-buffer, pH 4). After that, the MUC5AC antibody (1:100, MA5-12178, Invitrogen, USA) was applied in combination with the M.O.M Kit ImmPress (Vectorlabs, USA) as well as the diaminobenzidine (DAB) Substrate Kit (Vectorlabs, USA). Images were taken with an Axiostar microscope (Zeiss).

To assess collagen deposition in Sirius red-stained sections, six to nine airways per mouse were assessed. Analysis was performed using the Axiovision software 4.8 (Carl Zeiss, Germany). For quantification of goblet cell hyperplasia, PAS+ or MUC5AC+ cells in the airways were manually counted and values were normalized to the length of the basement membrane. Results were expressed as area normalized to the basement membrane perimeter.

For staining of ALI cultures, cells were fixated with paraformaldehyde and washed, then transwell membranes were isolated from the wells by cutting with a scalpel and embedded in paraffin. Five-micrometer sections were stained with Alcian blue. Twelve images were taken from different areas of the membrane and the number of goblet cells was counted manually and normalized to the length of the membrane.

Luminex measurements

Luminex measurements of lung homogenates were performed with Procarta Plex Mix&Match Mouse 6-plex (PPX-06-MXXGPZZ, Thermo Fisher Scientific, Vienna) and Luminex MagPix according to the manufacturer's instructions. Analysis of Luminex measurements was performed using Procarta-Plex-Analyst Software (Thermo Scientific, USA).

Statistical analysis

Statistical data analysis was performed using GraphPad Prism 5. Differences were determined by one- or two-way ANOVA and Tukey's or Bonferroni's post hoc test for multiple comparisons or unpaired two-tailed t test for comparison of two groups. Data are expressed as mean ± SEM. $*p < 0.05$ was considered significant.

DATA AVAILABILITY

All data associated with this study are present in the paper or the supplementary materials. Additional data related to this paper may be requested from the authors.

ACKNOWLEDGMENTS

We thank Anja Vöge (Uni Bochum) for excellent technical assistance with cell culture experiments and Gabriele König as well as Stefan Kehraus (Uni Bonn) for providing FR900359. We are also thankful to Natalie Katzmarski (Uni Bonn) for help with Luminex experiments and Gao Yuan Cao (The State University of New Jersey) for advice regarding ALI cultures and ELISA. The project was funded by the Deutsche Forschungsgemeinschaft (DFG, German Research Foundation)—FOR2372—D.W. (WE4461/2-1 and 2) and B.K.F. (FL-276/8-1 and 2).

AUTHOR CONTRIBUTIONS

J.M.D. performed *in vitro* and *in vivo* experiments and analyzed the data. M.M. performed stainings and *in vitro* experiments with NCI-H292 cells. A. Simon and A. Seidinger helped with *in vivo* experiments. C.K.-W. and R.A.P. supervised experiments with human lung slices. B.K.F. contributed to the writing of the manuscript. D.W. designed the study, analyzed data, and wrote the paper.

DECLARATION OF INTERESTS

The authors declare no competing interests.

SUPPLEMENTAL INFORMATION

Supplemental information can be found online at https://doi.org/10.1016/j.ymthe.2025.07.032.

REFERENCES

1. Livshits, G., and Kalinkovich, A. (2021). Receptors for pro-resolving mediators as a therapeutic tool for smooth muscle remodeling-associated disorders. Pharmacol. Res. *164*, 105340. https://doi.org/10.1016/j.phrs.2020.105340.
2. Domvri, K., and Porpodis, K. (2023). Targeting inflammation or remodeling in asthma? Is there a right way? Front. Med. *10*, 1241920. https://doi.org/10.3389/fmed.2023.1241920.
3. Hsieh, A., Assadinia, N., and Hackett, T.L. (2023). Airway remodeling heterogeneity in asthma and its relationship to disease outcomes. Front. Physiol. *14*, 1113100. https://doi.org/10.3389/fphys.2023.1113100.
4. Colak, Y., Afzal, S., Marott, J.L., Vestbo, J., Nordestgaard, B.G., and Lange, P. (2024). Type-2 inflammation and lung function decline in chronic airway disease in the general population. Thorax *79*, 349–358. https://doi.org/10.1136/thorax-2023-220972.
5. Hough, K.P., Curtiss, M.L., Blain, T.J., Liu, R.M., Trevor, J., Deshane, J.S., and Thannickal, V.J. (2020). Airway Remodeling in Asthma. Front. Med. *7*, 191. https://doi.org/10.3389/fmed.2020.00191.
6. Jude, J.A., and Panettieri, R.A., Jr. (2024). Bronchomotor tone imbalance evokes airway hyperresponsiveness. Expert Rev. Respir. Med. *18*, 835–841. https://doi.org/10.1080/17476348.2024.2419543.
7. Thomson, N.C. (2016). Addressing corticosteroid insensitivity in adults with asthma. Expert Rev. Respir. Med. *10*, 137–156. https://doi.org/10.1586/17476348.2016.1133304.
8. Huang, Y., and Qiu, C. (2022). Research advances in airway remodeling in asthma: a narrative review. Ann. Transl. Med. *10*, 1023. https://doi.org/10.21037/atm-22-2835.
9. Koziol-White, C., Gebski, E., Cao, G., and Panettieri, R.A., Jr. (2024). Precision cut lung slices: an integrated ex vivo model for studying lung physiology, pharmacology, disease pathogenesis and drug discovery. Respir. Res. *25*, 231. https://doi.org/10.1186/s12931-024-02855-6.
10. Matthey, M., Roberts, R., Seidinger, A., Simon, A., Schröder, R., Kuschak, M., Annala, S., König, G.M., Müller, C.E., Hall, I.P., et al. (2017). Targeted inhibition of G(q) signaling induces airway relaxation in mouse models of asthma. Sci. Transl. Med. *9*, eaag2288. https://doi.org/10.1126/scitranslmed.aag2288.
11. Busse, W.W. (2010). The relationship of airway hyperresponsiveness and airway inflammation: Airway hyperresponsiveness in asthma: its measurement and clinical significance. Chest *138*, 4S–10S. https://doi.org/10.1378/chest.10-0100.
12. Grainge, C.L., Lau, L.C.K., Ward, J.A., Dulay, V., Lahiff, G., Wilson, S., Holgate, S., Davies, D.E., and Howarth, P.H. (2011). Effect of bronchoconstriction on airway remodeling in asthma. N. Engl. J. Med. *364*, 2006–2015. https://doi.org/10.1056/NEJMoa1014350.
13. Rajavelu, P., Chen, G., Xu, Y., Kitzmiller, J.A., Korfhagen, T.R., and Whitsett, J.A. (2015). Airway epithelial SPDEF integrates goblet cell differentiation and pulmonary Th2 inflammation. J. Clin. Invest. *125*, 2021–2031. https://doi.org/10.1172/JCI79422.
14. Chen, G., Korfhagen, T.R., Xu, Y., Kitzmiller, J., Wert, S.E., Maeda, Y., Gregorieff, A., Clevers, H., and Whitsett, J.A. (2009). SPDEF is required for mouse pulmonary goblet cell differentiation and regulates a network of genes associated with mucus production. J. Clin. Invest. *119*, 2914–2924. https://doi.org/10.1172/JCI39731.
15. Wan, H., Kaestner, K.H., Ang, S.L., Ikegami, M., Finkelman, F.D., Stahlman, M.T., Fulkerson, P.C., Rothenberg, M.E., and Whitsett, J.A. (2004). Foxa2 regulates alveolarization and goblet cell hyperplasia. Development *131*, 953–964. https://doi.org/10.1242/dev.00966.
16. Zhu, L., An, L., Ran, D., Lizarraga, R., Bondy, C., Zhou, X., Harper, R.W., Liao, S.Y., and Chen, Y. (2019). The Club Cell Marker SCGB1A1 Downstream of FOXA2 is Reduced in Asthma. Am. J. Respir. Cell Mol. Biol. *60*, 695–704. https://doi.org/10.1165/rcmb.2018-0199OC.
17. Carr, R., 3rd, Koziol-White, C., Zhang, J., Lam, H., An, S.S., Tall, G.G., Panettieri, R. A., Jr., and Benovic, J.L. (2016). Interdicting Gq Activation in Airway Disease by Receptor-Dependent and Receptor-Independent Mechanisms. Mol. Pharmacol. *89*, 94–104. https://doi.org/10.1124/mol.115.100339.
18. Takeyama, K., Dabbagh, K., Lee, H.M., Agustí, C., Lausier, J.A., Ueki, I.F., Grattan, K.M., and Nadel, J.A. (1999). Epidermal growth factor system regulates mucin production in airways. Proc. Natl. Acad. Sci. USA *96*, 3081–3086. https://doi.org/10.1073/pnas.96.6.3081.
19. Gabazza, E.C., Taguchi, O., Tamaki, S., Takeya, H., Kobayashi, H., Yasui, H., Kobayashi, T., Hataji, O., Urano, H., Zhou, H., et al. (1999). Thrombin in the airways of asthmatic patients. Lung *177*, 253–262. https://doi.org/10.1007/pl00007645.
20. Amishima, M., Munakata, M., Nasuhara, Y., Sato, A., Takahashi, T., Homma, Y., and Kawakami, Y. (1998). Expression of epidermal growth factor and epidermal growth factor receptor immunoreactivity in the asthmatic human airway. Am. J. Respir. Crit. Care Med. *157*, 1907–1912. https://doi.org/10.1164/ajrccm.157.6.9609040.
21. Spector, C., De Sanctis, C.M., Panettieri, R.A., Jr., and Koziol-White, C.J. (2023). Rhinovirus induces airway remodeling: what are the physiological consequences? Respir. Res. *24*, 238. https://doi.org/10.1186/s12931-023-02529-9.
22. Leung, C., and Sin, D.D. (2022). Asthma-COPD Overlap: What Are the Important Questions? Chest *161*, 330–344. https://doi.org/10.1016/j.chest.2021.09.036.
23. Borchers, M.T., Biechele, T., Justice, J.P., Ansay, T., Cormier, S., Mancino, V., Wilkie, T.M., Simon, M.I., Lee, N.A., and Lee, J.J. (2003). Methacholine-induced airway hyperresponsiveness is dependent on Galphaq signaling. Am. J. Physiol. Lung Cell. Mol. Physiol. *285*, L114–L120. https://doi.org/10.1152/ajplung.00322.2002.
24. Kistemaker, L.E.M., Bos, S.T., Mudde, W.M., Hylkema, M.N., Hiemstra, P.S., Wess, J., Meurs, H., Kerstjens, H.A.M., and Gosens, R. (2014). Muscarinic M(3) receptors contribute to allergen-induced airway remodeling in mice. Am. J. Respir. Cell Mol. Biol. *50*, 690–698. https://doi.org/10.1165/rcmb.2013-0220OC.
25. Penn, R.B., Bond, R.A., and Walker, J.K.L. (2014). GPCRs and arrestins in airways: implications for asthma. Handb. Exp. Pharmacol. *219*, 387–403. https://doi.org/10.1007/978-3-642-41199-1_20.
26. Borchers, M.T., Justice, P.J., Ansay, T., Mancino, V., McGarry, M.P., Crosby, J., Simon, M.I., Lee, N.A., and Lee, J.J. (2002). Gq signaling is required for

allergen-induced pulmonary eosinophilia. J. Immunol. *168*, 3543–3549. https://doi.org/10.4049/jimmunol.168.7.3543.

27. Kistemaker, L.E.M., and Gosens, R. (2015). Acetylcholine beyond bronchoconstriction: roles in inflammation and remodeling. Trends Pharmacol. Sci. *36*, 164–171. https://doi.org/10.1016/j.tips.2014.11.005.

28. Schrage, R., Schmitz, A.L., Gaffal, E., Annala, S., Kehraus, S., Wenzel, D., Büllesbach, K.M., Bald, T., Inoue, A., Shinjo, Y., et al. (2015). The experimental power of FR900359 to study Gq-regulated biological processes. Nat. Commun. *6*, 10156. https://doi.org/10.1038/ncomms10156.

29. Annala, S., Feng, X., Shridhar, N., Eryilmaz, F., Patt, J., Yang, J., Pfeil, E.M., Cervantes-Villagrana, R.D., Inoue, A., Häberlein, F., et al. (2019). Direct targeting of Galpha(q) and Galpha(11) oncoproteins in cancer cells. Sci. Signal. *12*, eaau5948. https://doi.org/10.1126/scisignal.aau5948.

30. Mizuno, N., Kokubu, H., Sato, M., Nishimura, A., Yamauchi, J., Kurose, H., and Itoh, H. (2005). G protein-coupled receptor signaling through Gq and JNK negatively regulates neural progenitor cell migration. Proc. Natl. Acad. Sci. USA *102*, 12365–12370. https://doi.org/10.1073/pnas.0506101102.

31. Guo, J., Chung, U.I., Kondo, H., Bringhurst, F.R., and Kronenberg, H.M. (2002). The PTH/PTHrP receptor can delay chondrocyte hypertrophy in vivo without activating phospholipase C. Dev. Cell *3*, 183–194. https://doi.org/10.1016/s1534-5807(02)00218-6.

32. Gosens, R., Dueck, G., Rector, E., Nunes, R.O., Gerthoffer, W.T., Unruh, H., Zaagsma, J., Meurs, H., and Halayko, A.J. (2007). Cooperative regulation of GSK-3 by muscarinic and PDGF receptors is associated with airway myocyte proliferation. Am. J. Physiol. Lung Cell. Mol. Physiol. *293*, L1348–L1358. https://doi.org/10.1152/ajplung.00346.2007.

33. Krymskaya, V.P., Orsini, M.J., Eszterhas, A.J., Brodbeck, K.C., Benovic, J.L., Panettieri, R.A., Jr., and Penn, R.B. (2000). Mechanisms of proliferation synergy by receptor tyrosine kinase and G protein-coupled receptor activation in human airway smooth muscle. Am. J. Respir. Cell Mol. Biol. *23*, 546–554. https://doi.org/10.1165/ajrcmb.23.4.4115.

34. Cortijo, J., Mata, M., Milara, J., Donet, E., Gavaldà, A., Miralpeix, M., and Morcillo, E.J. (2011). Aclidinium inhibits cholinergic and tobacco smoke-induced MUC5AC in human airways. Eur. Respir. J. *37*, 244–254. https://doi.org/10.1183/09031936.00182009.

35. Zhu, W., Bi, M., Liu, Y., Wang, Y., Pan, F., Qiu, L., Guo, A., Lv, H., Yao, P., Zhang, N., and Wang, P. (2013). Thrombin promotes airway remodeling via protease-activated receptor-1 and transforming growth factor-beta1 in ovalbumin-allergic rats. Inhal. Toxicol. *25*, 577–586. https://doi.org/10.3109/08958378.2013.813995.

36. Zhou, S., Xiao, W., Pan, X., Zhu, M., Yang, Z., Zhang, F., and Zheng, C. (2014). Thrombin promotes proliferation of human lung fibroblasts via protease activated receptor-1-dependent and NF-kappaB-independent pathways. Cell Biol. Int. *38*, 747–756. https://doi.org/10.1002/cbin.10264.

37. Gosens, R., Nelemans, S.A., Grootte Bromhaar, M.M., McKay, S., Zaagsma, J., and Meurs, H. (2003). Muscarinic M3-receptors mediate cholinergic synergism of mitogenesis in airway smooth muscle. Am. J. Respir. Cell Mol. Biol. *28*, 257–262. https://doi.org/10.1165/rcmb.2002-0128OC.

38. Everman, J.L., Rios, C., and Seibold, M.A. (2018). Utilization of Air-Liquid Interface Cultures as an In Vitro Model to Assess Primary Airway Epithelial Cell Responses to the Type 2 Cytokine Interleukin-13. Methods Mol. Biol. *1799*, 419–432. https://doi.org/10.1007/978-1-4939-7896-0_30.

39. Boulet, L.P. (2003). Physiopathology of airway hyperresponsiveness. Curr. Allergy Asthma Rep. *3*, 166–171. https://doi.org/10.1007/s11882-003-0030-9.

40. Habre, W., Jánosi, T.Z., Fontao, F., Meyers, C., Albu, G., Pache, J.C., and Peták, F. (2010). Mechanisms for lung function impairment and airway hyperresponsiveness following chronic hypoxia in rats. Am. J. Physiol. Lung Cell. Mol. Physiol. *298*, L607–L614. https://doi.org/10.1152/ajplung.00222.2009.

41. Seidinger, A., Roberts, R., Bai, Y., Müller, M., Pfeil, E., Matthey, M., Rieck, S., Alenfelder, J., König, G.M., Pfeifer, A., et al. (2024). Pharmacological Gq inhibition induces strong pulmonary vasorelaxation and reverses pulmonary hypertension. EMBO Mol. Med. *16*, 1930–1956. https://doi.org/10.1038/s44321-024-00096-0.

42. Schlegel, J.G., Tahoun, M., Seidinger, A., Voss, J.H., Kuschak, M., Kehraus, S., Schneider, M., Matthey, M., Fleischmann, B.K., König, G.M., et al. (2021). Macrocyclic Gq Protein Inhibitors FR900359 and/or YM-254890-Fit for Translation? ACS Pharmacol. Transl. Sci. *4*, 888–897. https://doi.org/10.1021/acsptsci.1c00021.

43. Boesgaard, M.W., Harpsoe, K., Malmberg, M., Underwood, C.R., Inoue, A., Mathiesen, J.M., Konig, G.M., Kostenis, E., Gloriam, D.E., and Brauner-Osborne, H. (2020). Delineation of molecular determinants for FR900359 inhibition of G(q/11) unlocks inhibition of Galpha(s). J. Biol. Chem. *295*, 13850–13861. https://doi.org/10.1074/jbc.RA120.013002.

44. Patt, J., Alenfelder, J., Pfeil, E.M., Voss, J.H., Merten, N., Eryilmaz, F., Heycke, N., Rick, U., Inoue, A., Kehraus, S., et al. (2021). An experimental strategy to probe Gq contribution to signal transduction in living cells. J. Biol. Chem. *296*, 100472. https://doi.org/10.1016/j.jbc.2021.100472.

45. Cooper, P.R., Lamb, R., Day, N.D., Branigan, P.J., Kajekar, R., San Mateo, L., Hornby, P.J., and Panettieri, R.A., Jr. (2009). TLR3 activation stimulates cytokine secretion without altering agonist-induced human small airway contraction or relaxation. Am. J. Physiol. Lung Cell. Mol. Physiol. *297*, L530–L537. https://doi.org/10.1152/ajplung.00133.2009.

46. Vosen, S., Rieck, S., Heidsieck, A., Mykhaylyk, O., Zimmermann, K., Plank, C., Gleich, B., Pfeifer, A., Fleischmann, B.K., and Wenzel, D. (2016). Improvement of vascular function by magnetic nanoparticle-assisted circumferential gene transfer into the native endothelium. J. Control Release *241*, 164–173. https://doi.org/10.1016/j.jconrel.2016.09.024.

47. Simon, A., von Einem, T., Seidinger, A., Matthey, M., Bindila, L., and Wenzel, D. (2022). The endocannabinoid anandamide is an airway relaxant in health and disease. Nat. Commun. *13*, 6941. https://doi.org/10.1038/s41467-022-34327-0.

YMTHE, Volume *33*

Supplemental Information

Pharmacological Gq targeting prevents asthmatic airway remodeling

Jennifer M. Dietrich, Michaela Matthey, Annika Simon, Alexander Seidinger, Cynthia Koziol-White, Reynold A. Panettieri Jr., Bernd K. Fleischmann, and Daniela Wenzel

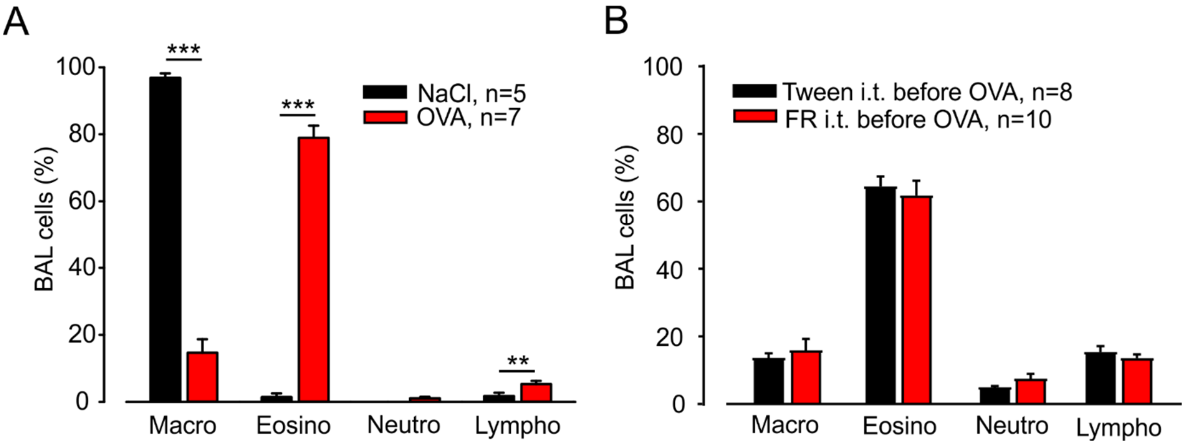

Figure S1. FR does not affects inflammatory cell counts in the OVA-induced mouse model of chronic asthma. A,B) Relative cell counts in BAL fluid for control and OVA-exposed Balb/c mice with chronic asthma (A), relative cell counts in BAL fluid for FR- or solvent-treated OVA-sensitized Balb/c mice (B), n represents the number of animals, ***P<0.001. A.B) Unpaired two-tailed student`s t-test.

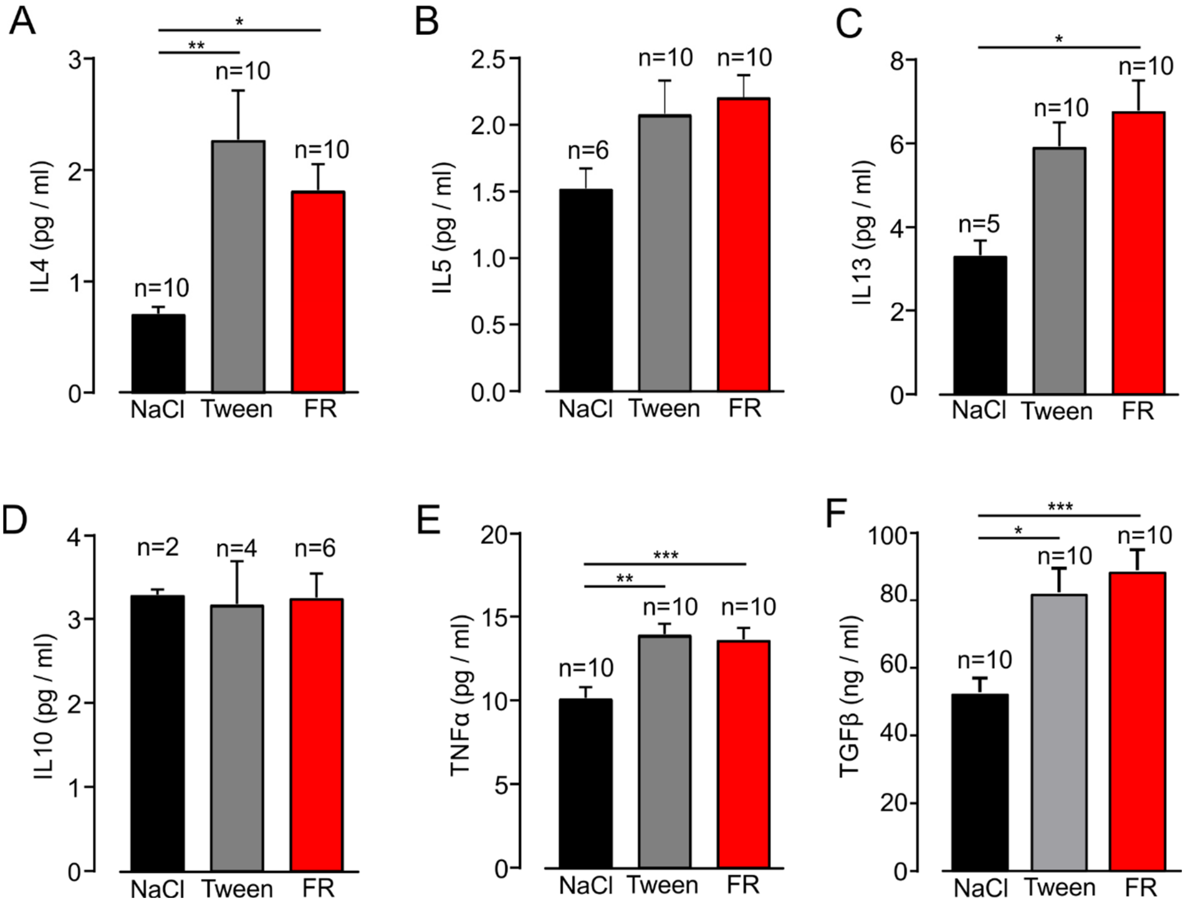

Figure S2. FR does not affect cytokine concentrations in the OVA-induced mouse model of chronic asthma. A-F) Cytokine levels of IL4 (A), IL5 (B), IL13 (C), IL10 (D), TNFα (E) and TGFβ (F) in lung homogenates of OVA-exposed animals after in vivo treatment with FR or the solvent compared to control animals, n represents the number of animals, *P<0.05, **P<0.01, ***P<0.001. A-F) One-way ANOVA, Tukey`s post hoc test.

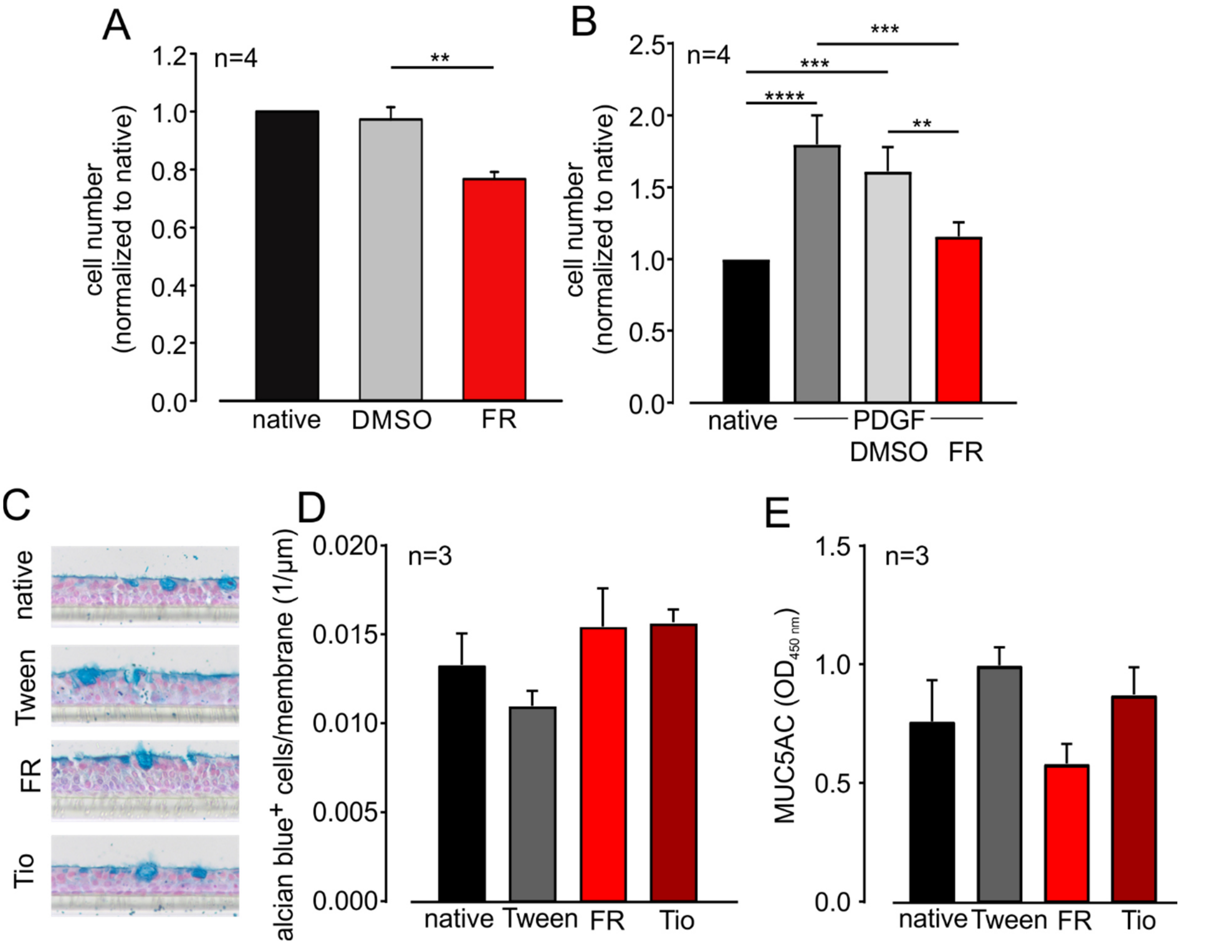

Figure S3. FR differentially modulates HFL1 cell growth and alcian blue+ goblet cell number of ALI cultures without pre-stimulation. A) Cell counts of HFL1 cells after treatment with FR or solvent. B) Cell counts of HFL1 cells after stimulation with PDGF BB (20 ng/ml) alone and treatment with FR (1 µM) or solvent DMSO for 5 days. C-E) Alcain blue staining of ALI cultures (C), number of alcian blue+ cells (D) and MUC5AC protein in the supernatant (E) after treatment with FR, tiotropium (Tio) or the solvent, n represents the number of different cell passages (A) or different wells (C,D), **P<0.01. A) One-way ANOVA, Tukey`s post hoc test.
